# Ontogenetic Shape Change in the Chicken Brain: Implications for Paleontology

**DOI:** 10.1371/journal.pone.0129939

**Published:** 2015-06-08

**Authors:** Soichiro Kawabe, Seiji Matsuda, Naoki Tsunekawa, Hideki Endo

**Affiliations:** 1 Gifu Prefectural Museum, Gifu, Japan; 2 The University Museum, The University of Tokyo, Tokyo, Japan; 3 Department of Anatomy and Embryology, School of Medicine, Ehime University, Ehime, Japan; 4 Department of Veterinary Anatomy, The University of Tokyo, Tokyo, Japan; University of Pennsylvania, UNITED STATES

## Abstract

Paleontologists have investigated brain morphology of extinct birds with little information on post-hatching changes in avian brain morphology. Without the knowledge of ontogenesis, assessing brain morphology in fossil taxa could lead to misinterpretation of the phylogeny or neurosensory development of extinct species. Hence, it is imperative to determine how avian brain morphology changes during post-hatching growth. In this study, chicken brain shape was compared at various developmental stages using three-dimensional (3D) geometric morphometric analysis and the growth rate of brain regions was evaluated to explore post-hatching morphological changes. Microscopic MRI (μMRI) was used to acquire in vivo data from living and post-mortem chicken brains. The telencephalon rotates caudoventrally during growth. This change in shape leads to a relative caudodorsal rotation of the cerebellum and myelencephalon. In addition, all brain regions elongate rostrocaudally and this leads to a more slender brain shape. The growth rates of each brain region were constant and the slopes from the growth formula were parallel. The dominant pattern of ontogenetic shape change corresponded with interspecific shape changes due to increasing brain size. That is, the interspecific and ontogenetic changes in brain shape due to increased size have similar patterns. Although the shape of the brain and each brain region changed considerably, the volume ratio of each brain region did not change. This suggests that the brain can change its shape after completing functional differentiation of the brain regions. Moreover, these results show that consideration of ontogenetic changes in brain shape is necessary for an accurate assessment of brain morphology in paleontological studies.

## Introduction

The organ shape of extant and extinct vertebrate animals changes during maturation or with increases in size, and the cranial part is of particular interest to many researchers [1–8]. The embryonic growth patterns of the brain and other organs in Aves are well described [9–11]. Early development of the chicken brain was first described by Kamon [12] and gross development of the chicken embryonic brain was described by Rogers [9]. Although many studies focused on the changes in brain volume and/or brain regions during maturation, including both embryonic and post-hatching growth [13–18], post-hatching changes in the shape of the avian brain due to growth are still poorly understood.

Recent paleoneurological studies have made positive progress and brains (cranial endocasts) of many species from various taxa have been analyzed from various angles using CT [19–28]. Nevertheless, interspecific or ontogenetic variations in the brains of fossil taxa have been rarely examined, since multiple specimens of one species or a close taxon are rarely obtained. Hence, paleontologists are usually forced to discuss the phylogeny or neurosensory development based on the brain morphology of an extinct species without consideration of the size or developmental stage of the specimen [24, 29–38]. Can we develop arguments on the brain morphology of extinct species without knowledge of the variation in the shape of the brain during growth? It is noted that brain shape changes considerably based on brain size in birds [39]. This indicates that brain size is important in assessing brain morphology in birds. That is, taking the developmental stage into account is essential when we evaluate the brain morphology of an extinct species. Smaller birds tend to have round, modern, avian-type brains, while larger birds show anteroposteriorly elongated, reptilian-type brains [39]. Given the pattern of shape change based on size, the brain of some species should change shape from the round to the anteroposteriorly elongated type during ontogeny. If that shape change is observed in a single species, then the brain changes its shape considerably during growth. We could incorrectly interpret two size differentiated brains from the same species as distinct species, leading to a misinterpretation of the morphology of the avian brain. Additionally, since the morphological characters that show ontogenetic change contain phylogenetic signals and can affect the results of phylogenetic analyses [40–43], it is imperative we know how avian brain shape changes during growth.

Since, in general, though not always the mass of the neural tissue of a particular region of the brain is correlated with the ability and/or sensory development of animals [44–48], comparisons of brain sizes of extant animals are used to estimate the degree of evolution of different sensory systems [44, 46]. Based on this principle, the relationships between the brain regional volume and sensory abilities of extinct birds and other animals were discussed in many studies [36, 49, 50]. As noted above, some previous studies measured the volumetric changes in brain regions based on growth in birds [13–18]. However, none investigated the covariation between the volume and shape of avian brains. In paleontological studies, the sensory and locomotor capability of extinct species are often assessed from the volume or area of brain regions [35, 37, 51]. However, all the primary data about brain that we can obtain from fossil specimens is external appearance of entire brain, and we had to judge the degree of development in brain regions from external information of brain.

The aims of this study were to quantitatively describe developmental shape changes in the chicken brain and to compare the growth pattern of each brain region with changes in brain shape during development. We also investigated the relationship between the volume and shape of brain regions. Since the relationship between the volume and shape of avian brains has not yet been investigated, it is unclear whether we can assess the cognitive abilities of extinct birds from the appearance of the brain. To achieve these aims, brain shape was compared among various developmental stages in the chicken using 3D geometric morphometric analysis and the growth rates of brain regions (telencephalon, diencephalon and mesencephalon, cerebellum, and myelencephalon) were evaluated using simple regression analysis to explore post-hatching morphological changes in the chicken brain.

## Materials and Methods

### Chicken eggs

This study was carried out in strict accordance with the recommendations of the Guidelines of the Animal Care Committee of Ehime University. The protocol was approved by the Animal Care Committee of Ehime University (Permit Number: 05A-27-10). The broiler eggs were incubated at 38°C in a rocking, humidified incubator for 24 days. They were then manually turned several times per day. Forty-four eggs hatched in August 2011 and the chicks were raised for a maximum of 118 days (Table 1).

**Table 1:** Volume of the brain, including eyes, brain, brain and eyes, telencephalon, dien- and mesencephalon, and cerebellum of sampled specimens.

Specimen ID	Day	Body weight (g)	Eyes (mm^3^)	Telencephalon (mm^3^)	Dien- Mesencephalon (mm^3^)	Cerebellum (mm^3^)	Myerencephalon (mm^3^)	Whole brain (mm^3^)	Eyes + Brain (mm^3^)
#1	119	5300	-	2216.875	994.5811	588.4555	201.2268	4001.139	-
	20	282	-	685.255	343.3328	175.4841	55.11907	1259.191	-
#2	26	752	1533.867	1286.919	477.0751	273.0119	81.52103	2118.527	3652.394
	15	286	1095.275	737.231	362.7126	265.9438	115.0294	1480.917	2576.192
#3	34	1140	1606.663	1229.209	617.3489	331.4657	137.6827	2315.706	3922.369
	16	264	1234.698	799.3646	391.8207	242.8153	107.762	1541.763	2776.461
#4	22	436	1334.28	1176.198	429.1162	275.672	79.7655	1960.752	3295.032
	17	286	-	906.6819	431.7303	237.4183	82.42563	1658.256	-
	7	88	-	559.0485	262.747	146.744	62.24085	1030.78	-
#5	91	5500	-	2240.134	1042.686	711.5726	204.6842	4199.077	-
	15	328	-	794.4812	441.9108	303.8449	92.30719	1632.544	-
	11	202	987.7967	739.7763	334.4708	228.5717	108.13	1410.949	2398.745
	2	48.2	-	517.3658	257.5618	145.2118	49.6468	969.7863	-
#6	34	1010	-	1352.357	491.6407	405.451	85.3164	2334.765	-
	16	312	1092.37	722.5275	334.2332	227.7131	111.8327	1396.306	2488.676
#7	116	7400	5142.16	2312.18	1126.583	744.4218	340.458	4523.643	9665.802
	13	268	1335.116	628.5874	355.1002	201.8324	115.7501	1301.27	2636.386
	11	208	964.1316	598.1609	335.3371	218.9968	109.4562	1261.951	2226.083
	1	51	-	489.6003	265.8774	148.6873	54.2146	958.3795	-
#8	20	470	1286.858	993.4312	427.0233	273.4105	96.30122	1790.166	3077.024
	7	132	795.7233	582.1387	289.1413	200.3375	109.3489	1180.966	1976.69
	2	56.6	595.4765	510.5206	243.49	143.5814	80.04221	977.6342	1573.111
	1	55.2	711.1449	541.3035	249.6146	145.7596	73.52684	1010.204	1721.349
#9	35	1380	1971.116	1628.029	569.2136	358.8872	153.7968	2709.926	4681.043
#10	38	1910	1891.167	1449.371	657.1665	407.3291	143.8615	2657.728	4548.895
#11	40	1450	1898.25	1455.067	571.1915	420.9978	155.9203	2603.176	4501.427
#12	86	4960	5542.183	2395.319	967.382	629.653	308.4215	4300.775	9842.958
#13	94	6000	5486.382	2397.726	1008.74	593.4998	251.9072	4251.873	9738.255
#14	15	258	1259.551	811.0093	411.7908	238.2079	133.4894	1594.497	2854.049
#15	46	2160	2897.04	1696.694	698.5325	483.3612	206.9533	3085.541	5982.58
#16	20	450	1274.362	898.3335	392.0201	244.0494	137.3454	1671.748	2946.111
#17	42	1580	1931.73	1756.03	724.75	611.26	223.66	3315.7	5247.43
#18	26	698	1563.181	1176.528	474.415	292.4377	143.0413	2086.422	3649.603
#19	119	5200	2386.089	1864.205	854.2535	628.6717	124.7347	3471.865	5857.954
#20	32	1060	1603.704	1326.507	450.4125	348.0704	146.6827	2271.672	3875.377
#21	84	4700	-	1991.776	1060.793	710.4534	115.7807	3878.803	-
#22	13	124	793.0706	553.0153	224.7003	153.9424	78.00996	1009.668	1802.739
#23	30	780	1765.857	1249.248	526.8356	304.4888	124.0984	2204.671	3970.528
#24	33	990	1960.522	1389.039	576.2204	381.7245	135.6895	2482.673	4443.195
#25	24	562	-	946.8904	365.1811	206.7157	66.54919	1585.336	-
#26	8	150	834.3523	655.7941	327.9623	192.7327	92.4835	1268.973	2103.325
#27	14	292	1228.236	646.4417	378.0371	176.2891	84.07384	1284.842	2513.077
#28	12	250	-	755.5146	396.7193	235.7088	87.64622	1475.589	-
#29	5	66.2	723.5254	553.1523	260.8034	162.1793	48.1637	1024.299	-
#30	20	460	-	950.5855	406.9995	249.0784	77.49634	1684.16	-
#31	16	300	-	674.9442	298.2716	135.4672	45.2145	1153.897	-
#32	10	148	-	538.2504	264.4796	168.3853	71.91544	1043.031	-
#33	18	342	-	705.9381	301.3073	172.8546	49.79115	1229.891	-
#34	30	950	1726.883	1230.681	518.3032	293.258	143.6545	2185.896	3912.779
#35	32	970	-	1205.705	528.6908	421.8794	205.3434	2361.618	-
#36	22	560	1587.874	1168.908	478.7617	251.2326	89.5934	1988.495	3576.369
#37	28	686	-	1145.948	472.1611	340.159	85.89069	2044.159	-
#38	36	1160	2038.11	1372.135	509.3799	337.3302	114.0405	2332.886	4370.996
#39	35	1390	2272.37	1710.025	570.8695	434.0684	216.6279	2931.591	5203.96
#40	75	3000	3159.385	2212.237	938.9714	739.5308	177.9679	4068.707	7228.092
#41	30	940	-	1353.783	502.8561	349.8643	117.8582	2324.361	-
#42	4	68	589.8324	510.5782	276.2558	145.01	62.36681	994.2108	1584.043
#43	24	606	-	1052.859	418.9663	240.1014	90.32935	1802.256	-
#44	28	714	-	1183.389	516.2258	318.3184	97.02183	2114.955	-

### μMRI and image acquisition

**Fig 1:**
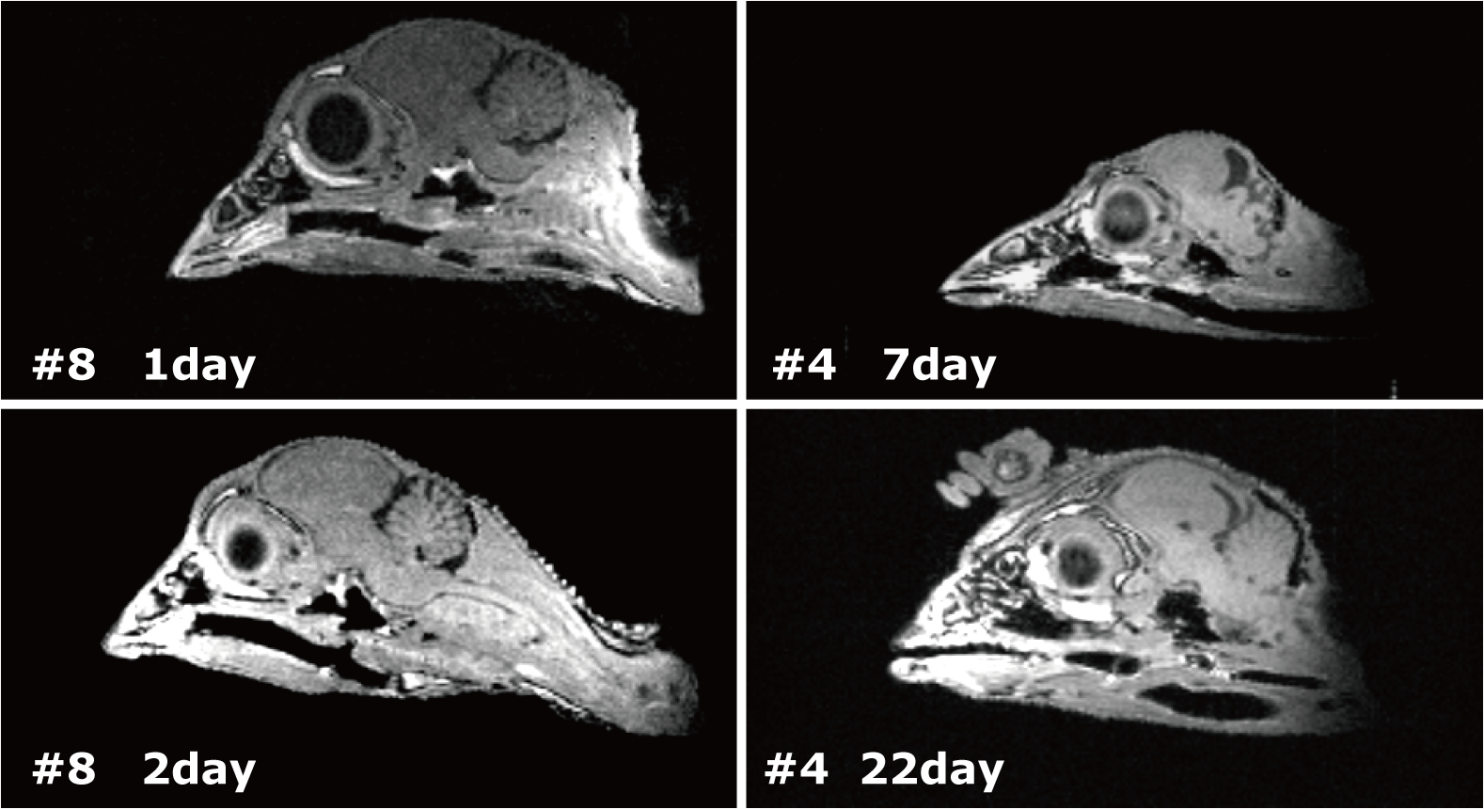
MRI images acquired by μMRI, MRmini SA (MRTechnology, Inc).

μMRI is a non-invasive method that allows for the differentiation of major brain regions from each other. Furthermore, it permits repeated viewing of the same living specimen. Hence, μMRI was used to acquire in vivo volumetric data and data from post-mortem chicken brain and eyes. μMRI was performed using a 1.5-T, MRmini SA (MRTechnology, Inc) at Ehime University (Toon, Japan). Both 30 and 38.5 mm diameter RF coils were used based on the size of the chicken. During scanning, the birds were anesthetized with isoflurane using a gas anesthesia system. When they reached the opening of the MRI apparatus, they were beheaded, and only the heads were scanned by MRI. That is, the decapitation had been at the final growth stage in each development. Images used to create the 3D chicken brain model were recorded using 3D spin-echo sequence mode acquisition, with a data matrix of 512 × 256 × 128 points (Fig 1).

Identified brain regions (telencephalon, diencephalon and mesencephalon, cerebellum, and myelencephalon) and eyes were manually labeled using the segmentation tools available in the Amira visualization software (v 5.3.2, Mercury Computer Systems, San Diego, CA, USA). The 3D models were created and brain volumes were calculated by using Amira visualization software. Details of the methods used to prepare and examine the 3D models were previously described by Corfield et al. [52].

### 3D geometric morphometrics

**Fig 2:**
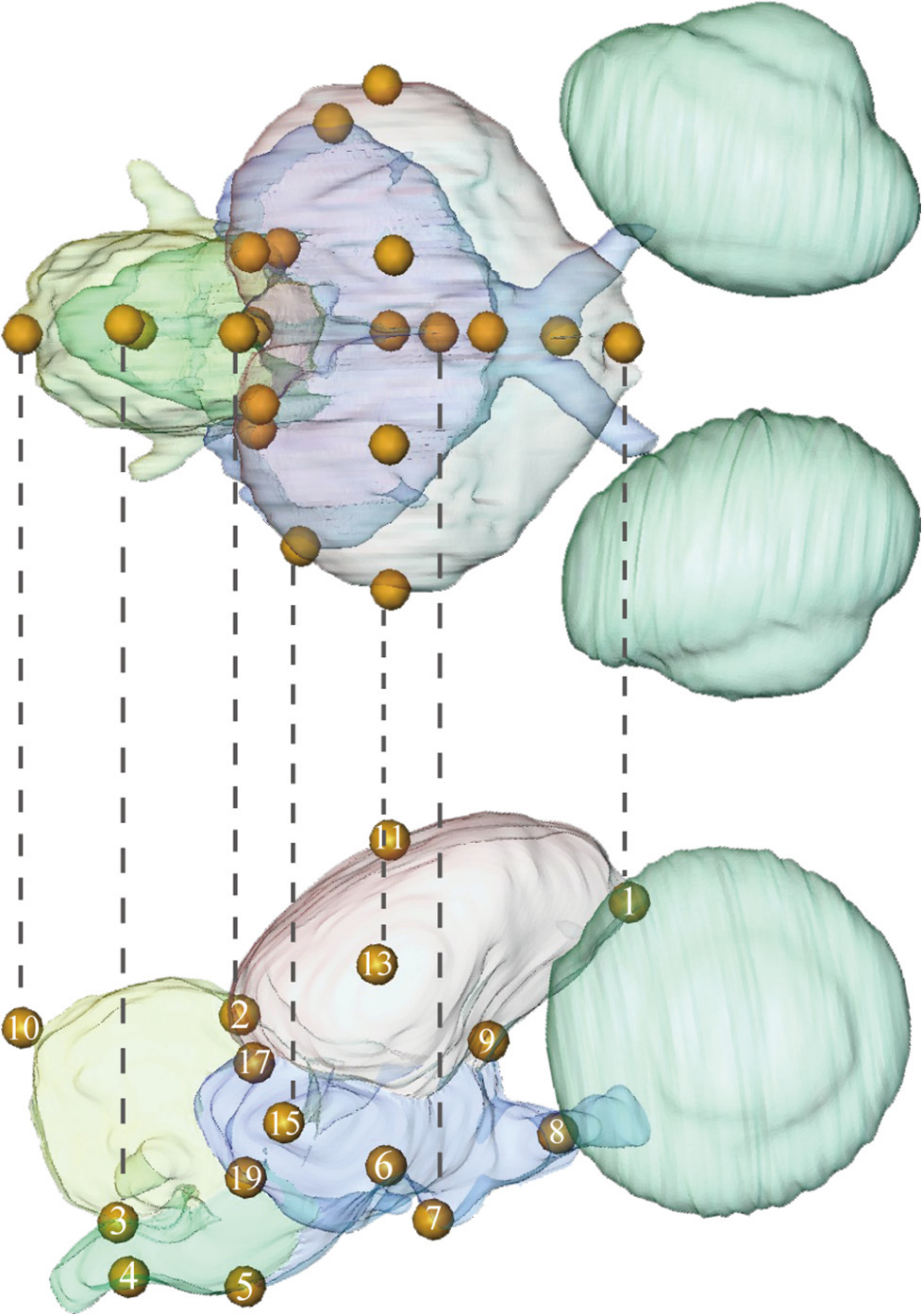
The three-dimensional brain landmarks used for shape analysis shown in dorsal (upper) and right lateral (lower) views.

**Fig 3:**
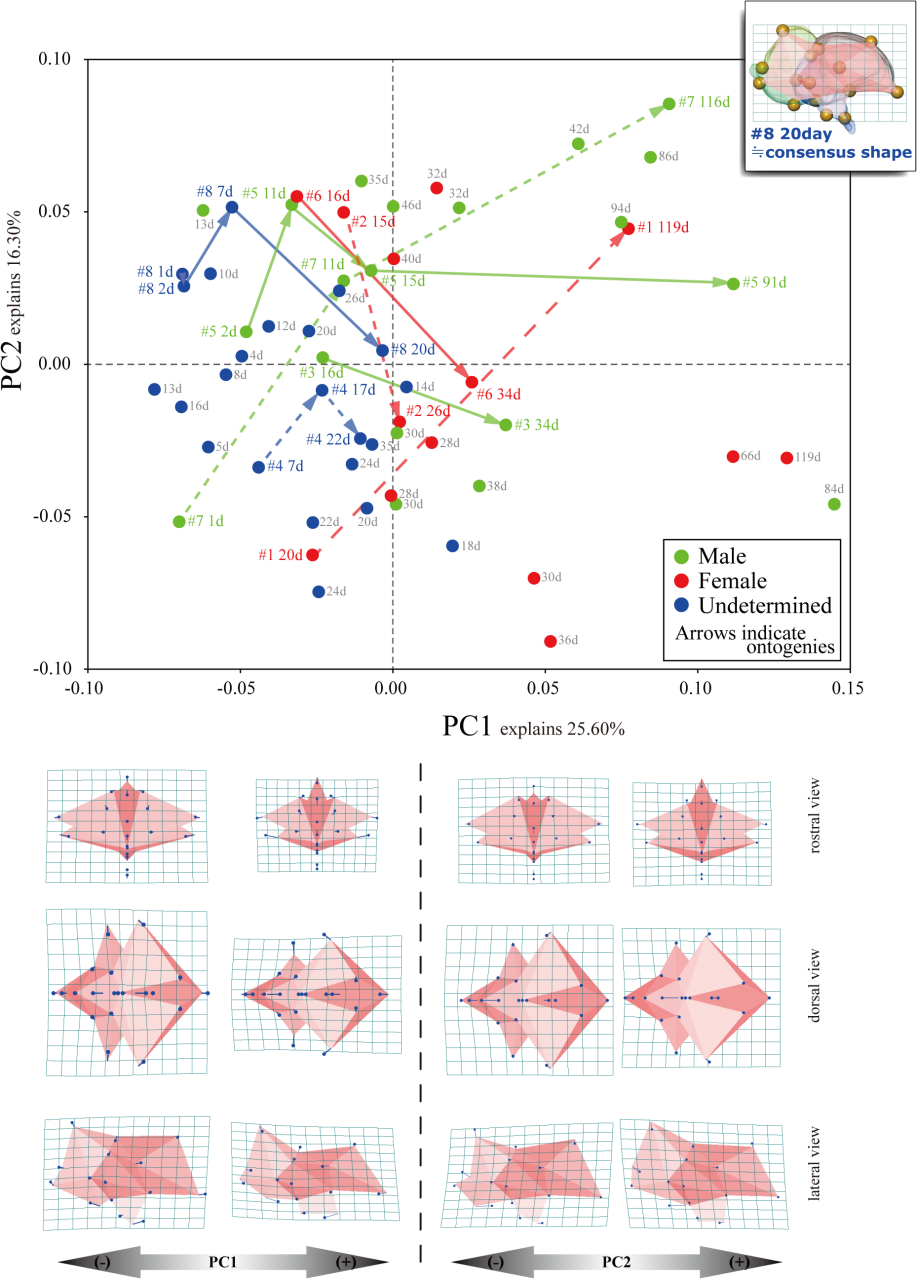
Ontogenetic shape variations (#1 to #8) (not to scale).

The 3D coordinates from 20 homologous landmarks of the brain were digitized (Table 2, Fig 2) from 43 specimens using Amira. Five of them (#1 to #8; Fig 3) were scanned several times at different developmental stages. The resulting 3D coordinate data set was subjected to generalized Procrustes analysis (GPA; [53]) using the MorphoJ software package [54]. In GPA, distances between homologous landmarks are minimized by translating, rotating, and scaling all objects to a common reference. That is, the effects of size, position, and orientation are eliminated so that remaining data reflect shape variation (Procrustes shape coordinates). Information on the absolute size of the specimen is preserved as centroid size (CS), which is calculated as the square root of the sum of squared distances of landmarks from their centroids [55].

**Table 2:** Landmarks used (Fig 2) and anatomical descriptions (refer to Fig 2) for profiled anatomical structures.

Number	Anatomical description
1	Median anterior tip of the telencephalon
2	Median junction between the telencephalon and cerebellum
3	Median dorsal point of the foramen magnum
4	Median ventral point of the foramen magnum
5	Median junction between the mesencephalon and myelencephalon
6	Median junction between the hypophysis and mesencephalon
7	Median ventral tip of the hypophysis
8	Median point where the two optic nerves intersect
9	Median junction between the telencephalon and mesencephalon
10	Perpendicular at midpoint between landmarks 2 and 3 to dorsal margin of cerebellum in lateral view
11, 12	Perpendicular at midpoint between landmarks 1 and 2 to dorsal margin of telencephalon in lateral view, right and left
13, 14	Most lateral point of the widest part of the telencephalon, right and left
15, 16	Most lateral point of the widest part of the floccular lobe, right and left
17, 18	Intersection of the telencephalon, cerebellum, and optic lobe, right and left
19, 20	Intersection of the cerebellum, myelencephalon, and optic lobe, right and left

### Principal component analysis (PCA)

**Fig 4:**
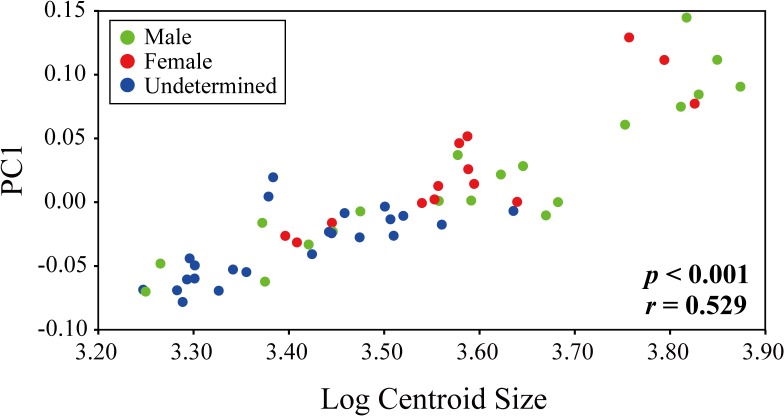
PCA and variation in brain shape for each principal component (PC) score.

**Fig 5:**
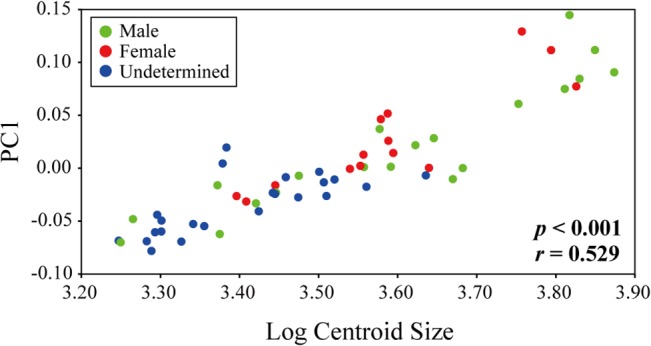
Regression analysis of PC1 on log centroid size.

**Fig 6:**
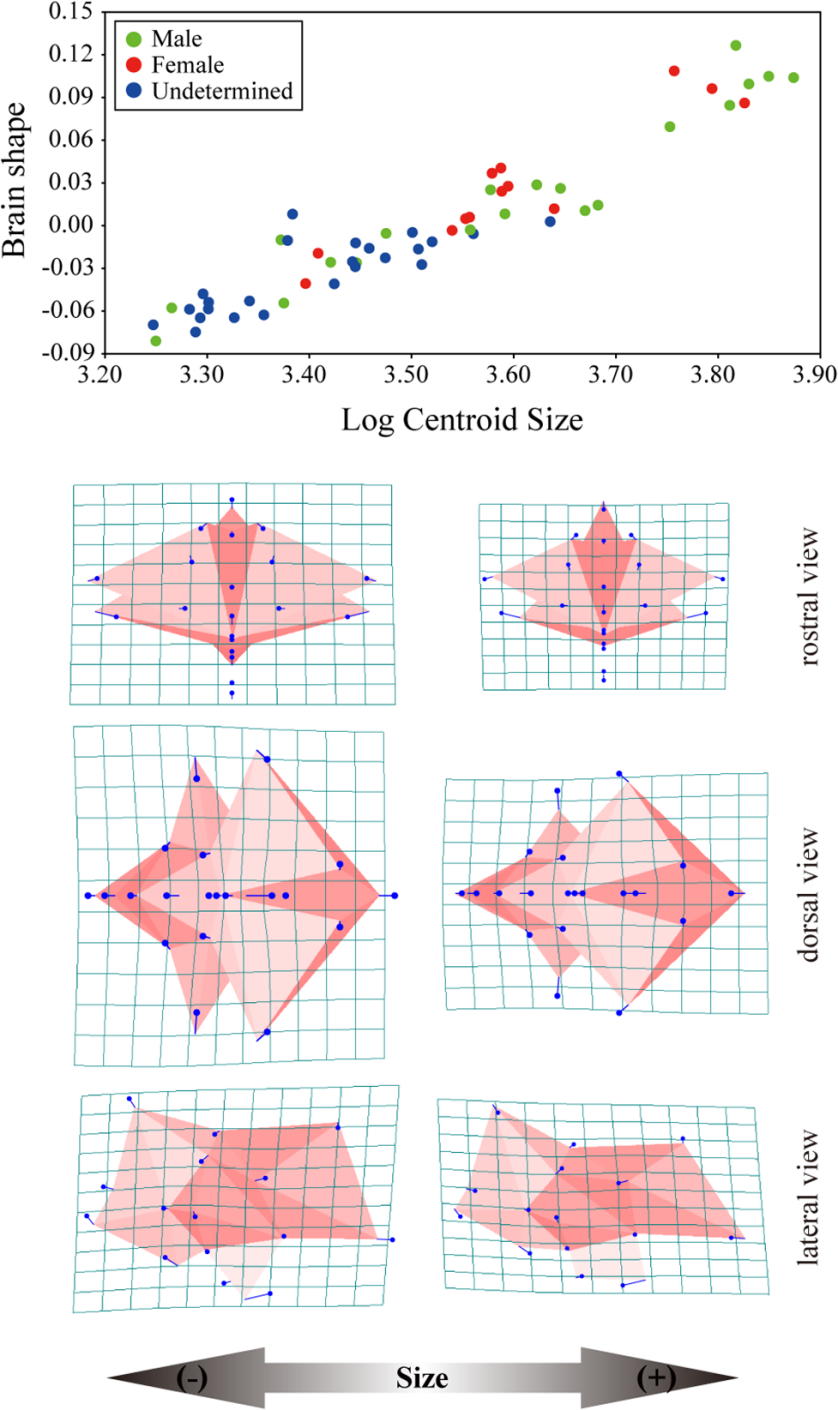
Multivariate regression of brain shape coordinates on log centroid size.

Procrustes shape coordinates were subjected to PCA to explore the patterns of major variation among chicken brains at various developmental stages (sizes) (Fig 4). The proportion of total variance contributed by each PC and the cumulative total for the first 10 PCs from the PCA are provided in Table 3. PCA was performed using MorphoJ, and Morphologika was used to illustrate the 3D profiles [55]. The scores of specimens along the PC axes and log CS were subjected to correlation and regression analyses to examine the effect of aging, namely, the effect of increasing size on brain shape (Table 4, Fig 5). Regression analysis was performed to determine whether size alone was responsible for the differences in shape observed along the PC axes. Regression coefficients are vectors representing the correlations between changes in shape and size. To explore how shape varies with growth, multivariate regressions of brain shape onto brain volume were performed (Fig 6). Statistical significance was tested using a permutation test against the null hypothesis of size independence.

**Table 3:** Eigenvalues and explanatory proportion for the first 10 principal components (PCs) from the principal component analysis.

PC	Eigenvalue	Proportion (%)	Cumulative (%)
1	0.00280665	23.383	23.383
2	0.00186082	15.503	38.885
3	0.00103005	8.581	47.467
4	0.00094377	7.863	55.33
5	0.0008114	6.76	62.089
6	0.0006503	5.418	67.507
7	0.00052655	4.387	71.894
8	0.00045568	3.796	75.69
9	0.0004347	3.622	79.312
10	0.00033285	2.773	82.085

**Table 4:** Results of the correlation (*r*) and regression (*R* ^2^, *P*) analysis between the PC scores and log centroid size (CS).

		PC1	PC2	PC3	PC4
**CS**	***r***	0.5259	0.2128	0.2296	-0.0224
	***R*** ^***2***^	0.2766	0.0453	0.0527	0.0005
	***P***	<0.0001	0.1709	0.0313	0.9847

### Growth rate

**Fig 7:**
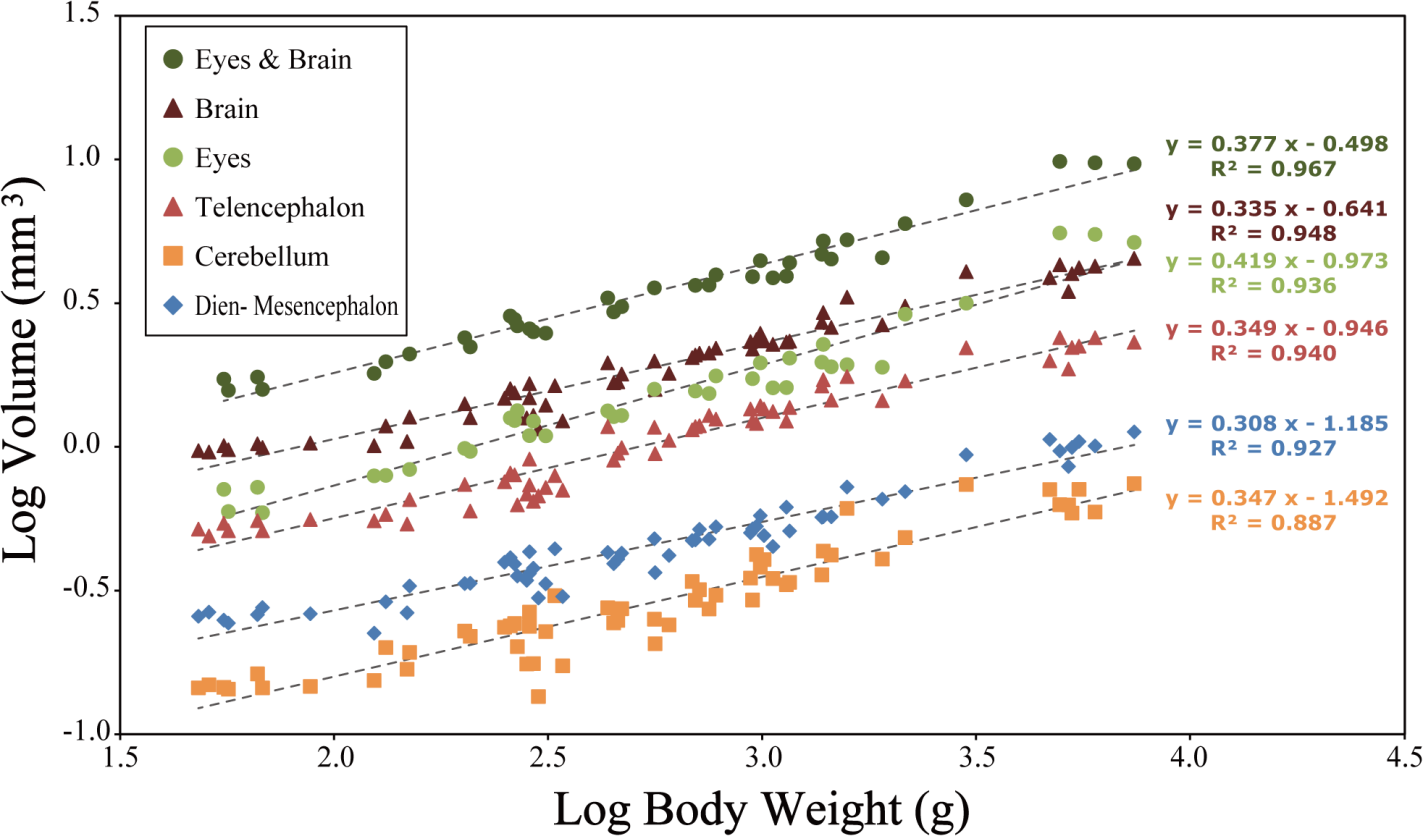
Relationship between body weight and eyes + brain (dark green circles), whole brain volume (brown triangles), eyes (light green circles), and brain regions (telencephalon, red triangles; cerebellum, yellow squares; diencephalon and mesencephalon, blue diamonds).

To explore the relationship between the growth of the brain and shape, the logarithmic volume of each brain region (telencephalon, diencephalon and mesencephalon, cerebellum, and myelencephalon) and both eyes were regressed on the log of body weight (Fig 7). Chickens were weighed on a scale before MRI scanning.

## Results

The first three PCs from the PCA analysis accounted for 47.47% of the total shape variation and provided a reasonable approximation of the total variation in shape (Table 3; Fig 4). Since PC1 and PC2 were the only PC axes that accounted for more than 10% of the variance (Table 3; Fig 4), the following descriptions and discussions will be based on these two PCs.

The telencephalon rotates caudoventrally with increasing PC1 score (Fig 4). This change in shape leads to a relative caudodorsal rotation of the cerebellum and myelencephalon. With increasing PC1, all brain regions elongate rostrocaudally, which results in a more slender brain shape. In particular, the optic nerves elongate rostrodorsally. This extension of the optic nerve indicates that the orbit has a tendency to be located more caudally.

The PC2 axis mainly corresponds to lowering of the cerebellum (Fig 4). The telencephalon and myelencephalon change their posterior orientation under the influence of the rotation of the cerebellum.

Since PC1 explained a considerable degree of the shape variation, the shape change accompanying brain growth can be summarized as shape change with increasing PC1 score (Fig 4). The correlation between the PC1 score and log centroid size (CS) was significant (*r* = 0.5289, *P* < 0.001; Table 4, Fig 5). Thus, brain shape along the first dimension was affected by increases in size (Fig 5). The multivariate regression analysis of shape against size explained 20.20% of the shape variation and it revealed that the correlation between shape and size is significant (*p* < 0.001; Fig 6). Comparing larger brains to smaller brains (lower right to upper left in Fig 6), the length of the telencephalon and myelencephalon varies quite substantially. In addition, the optic nerve tends to elongate and rotate rostrodorsally with increasing brain size. These shape changes correspond to positive changes along the PC1 axis (Figs 3 and 5). That is, the ontogenetic changes of the brain are accurately reflected in these two patterns of shape change.

The growth rates for each brain region were constant and the slopes of the growth formula were parallel (Fig 7). However, eye size was relatively large, indicating that the eye exhibits tachyauxesis against the entire brain (Fig 7).

## Discussion

### Shape change

Changes in brain shape during post-hatching development in chickens can be described by positive shape changes in the PC1 axis and multivariate regression. The dominant pattern of shape change was as follows: (1) caudoventral rotation of the telencephalon, (2) caudodorsal rotation of the cerebellum and myelencephalon, (3) rostrocaudal elongation of the entire brain, and (4) extension of the optic nerve in the rostrodorsal direction (Figs 3 and 5). We explored the relationship between brain shape and brain size in Aves using various taxa (60 species from 22 orders) and discovered the dominant allometric shape change [39]. Brain posture and relative brain length dramatically change based on brain size in Aves [39]. The ontogenetic pattern of shape change described above (1)–(3) corresponds to interspecific shape changes with increasing brain size [39]. That is, the intraspecific and interspecific ontogenetic changes in brain shape with increasing size display similar patterns.

The anteriorly elongated optic nerve orients the eyeball and orbit more rostrally. The covariation pattern between brain shape and orbital shape was also discussed in Kawabe et al. [39]; the rostrally elongated orbit has a rostrocaudally elongated brain. In light of the covariation between brain shape and orbital shape described in Kawabe et al. [39], the orbital shape changes from a round type to an elongated type during growth.

Although PC1 explained most of the variation in shape change, the contribution from PC2 was relatively high (Fig 4) and was concentrated mainly in the cerebellum. The shape of the cerebellum is relatively variable due to factors other than size. Plots of each male and female were not deflected to one side of the PC2 axis (Fig 4). Hence, sexually dimorphic variation among brain shape in chickens is vanishingly small. Since the PC2 score did not significantly correlate with size (Table 4) and did not reflect sexual dimorphism (Fig 4), the shape change based on the PC2 axis reflects individual variability other than ontogeny and sexual dimorphism.

### Size change

The chicken is precocial bird [56–58] and negative allometry was found in the brains of the broiler chickens used in this study (Fig 7). Previous studies also found negative allometry in many precocial bird species, including some domestic chickens [17, 18, 59–63]. This negative allometry is due to slow post-hatching brain growth [63]. Although the brains of the broiler chickens had a slope (exponent of 0.335) similar to many precocial birds, the slope of the birds in this study is relatively low compared to mallards, ducks [61, 63], and other precocial birds [59, 60, 61]. It is thought that the rapid growth and heavy body of the broiler [64, 65, 66] lowers the allometric slope of the brain and each brain region. However, compared to white leghorns [17], and other domestic chickens [18], the allometric slope of the broiler is relatively high for the brain and each brain region, even considering the slight differences in the brain regional borders between previous works and this study. The slopes of the brain and each brain region are ~0.25 or lower in every domestic chicken [17, 18], except the broiler. This indicates that the brain and each brain region in the boiler grows relatively rapidly compared to other chickens, but slowly compared to many other precocial birds. Further studies are needed to determine the effect of such differences in growth rate on brain morphology in various domestic chickens and other avian taxa.

The similar brain growth rate in the brain regions of chickens is due to differences in growth pattern, i.e. the differences between the precocial and altricial patterns. One of the most striking observations is the substantial difference in chick and adult brain sizes of altricial and precocial birds [62]. The difference in brain volume between precocial and altricial species is well known [13, 62, 67, 68]. All precocial species and some altricial species almost complete brain growth during embryogenesis, hatch with relatively large brains, and undergo little brain growth development from chick to adult [62]. On the other hand, hence the brains of many altricial birds considerably increase in size after hatching compared with those of precocial birds [63], the brain regions of altricial birds are supposed to develop at a different rate after hatching. It is thought that functional differentiation of each brain region in chickens is nearly complete by hatching and this leads to similar growth rates among each brain region.

There was a significant positive relationship between eye size and body size (Fig 7). Additionally, it was found that eyes grow at a faster rate than the brain. Garamszegi et al. [69] calculated the allometric relationship of eye size and brain size to body size from 141 and 159 bird species, respectively. Interspecific allometric equations for eye size and brain size on body mass showed that the avian eye exhibits tachyauxesis against the brain [69] and are consistent with our results from the ontogenetic analysis. This indicates that the rapid growth of the chicken eye is a simple matter of allometric relationships, not functional development through growth.

### Relationships between shape and size

The shape of the brain and each brain region changed considerably; however, the volume ratio of each brain region did not change (Fig 7). That is, the brain can change its shape without variation in the proportional and relative sizes of brain regions in post-hatching growth. Distinct functions are localized within the brain [70] and the relative size of each brain region is indicative of their importance in the life of the animal [44]. Therefore, it would appear that functional changes of the brain already modestly advanced at hatching, since the relative sizes of the brain regions do not change throughout its life. In other words, the brains of chickens change shape after measurable functional differentiation of the brain regions, although we could not identify changes in the morphological characters that relate to function based on the ontogenetic shape differences.

### Implications for paleontology

As discussed above, we assumed that functional differentiation of the brain in precocial birds, including chickens, is nearly complete by hatching, since the ratios of the brain regions were constant throughout growth. Many non-avian dinosaurs, which are avian ancestors and relatives, are thought to have been precocial [71, 72] and differentiation of their brains is probably complete at hatching, judging from the developing pattern. Thus, the brains of dinosaurs should show roughly similar ontogenetic changes to extant precocial birds, including chickens. The growth process of non-avian theropods leads to a more rostrocaudally elongated brain; indeed, this has been observed in some fossil specimens [73]. Hence, when we compare brain endocasts of the same extinct species, we have to pay due consideration to allometric relationships. Otherwise, we could develop fallacious arguments about fossil animals.

We also concluded that it is difficult to recognize functional development from brain and eye shape in the development of chickens. Brain regions showed no relative volumetric change, though their shape changed considerably. Many studies have discussed the sensory and locomotor abilities of extinct animals by assessing the relative size of individual brain regions based on their appearance in endocasts of fossil specimens [24, 29–38]. However, according to our results, it is not necessarily appropriate to suggest that the relative sizes of brain regions can be determined from external morphology. Since the interior boundaries of the brain regions are indefinable from extinct brain endocasts, we cannot determine the exact value of the regional volume in the brain of an extinct species. Despite these limitations, paleontologists have attempted to calculate the volume or area of the brain regions of extinct avian species [51, 74, 75]. Although we need to establish whether the volume of brain regions significantly correlates with area, assessing the brain morphology of extinct species in these quantitative ways has enormous implications for paleoneurological studies.

## Conclusions

We assessed the ontogenetic changes in the brain shape of chickens using μ MRI. The dominant pattern of shape change was as follows: (1) rostrodorsal rotation of the telencephalon, (2) caudoventral rotation of the cerebellum and myelencephalon, (3) rostrocaudal elongation of the entire brain, and (4) extension of the optic nerve in a rostroventral direction. The pattern of these shape changes corresponds to interspecific shape changes due to increases in size. The interspecific and ontogenetic shape changes with increasing size exhibit similar patterns. Not all of the shape variation can be explained by size. The variations that cannot be explained by size are concentrated in the cerebellum. Changes in brain shape were also observed with no change in the ratio of individual brain regions. Growth of brain regions at the same rate as other regions is due to the nearly complete functional differentiation of the brain at hatching. Therefore, we concluded that it is difficult to recognize functional development from brain and eye shape in the development of chickens. A detailed analyses of the brain morphology in other taxon including palaeognathous birds is needed to address the universal rule of the ontogenetic shape change in avian brain, and this study is the starting point for understanding changes in brain shape during post-hatching development in birds. This work, however, provides an important particular case of the ontogenetic shape change, and is critical for future work on the avian brain morphology.
