# Correction: Ontogenetic Shape Change in the Chicken Brain: Implications for Paleontology

**DOI:** 10.1371/journal.pone.0133456

**Published:** 2015-07-20

**Authors:** Soichiro Kawabe, Seiji Matsuda, Naoki Tsunekawa, Hideki Endo

The correct Fig 3 does not appear in the article. The correct Fig 4 is erroneously published as Fig 3, and the correct Fig 5 is published as both Fig 4 and Fig 5. The authors have provided the correct Fig 3 and Fig 4 here.

**Fig 3:**
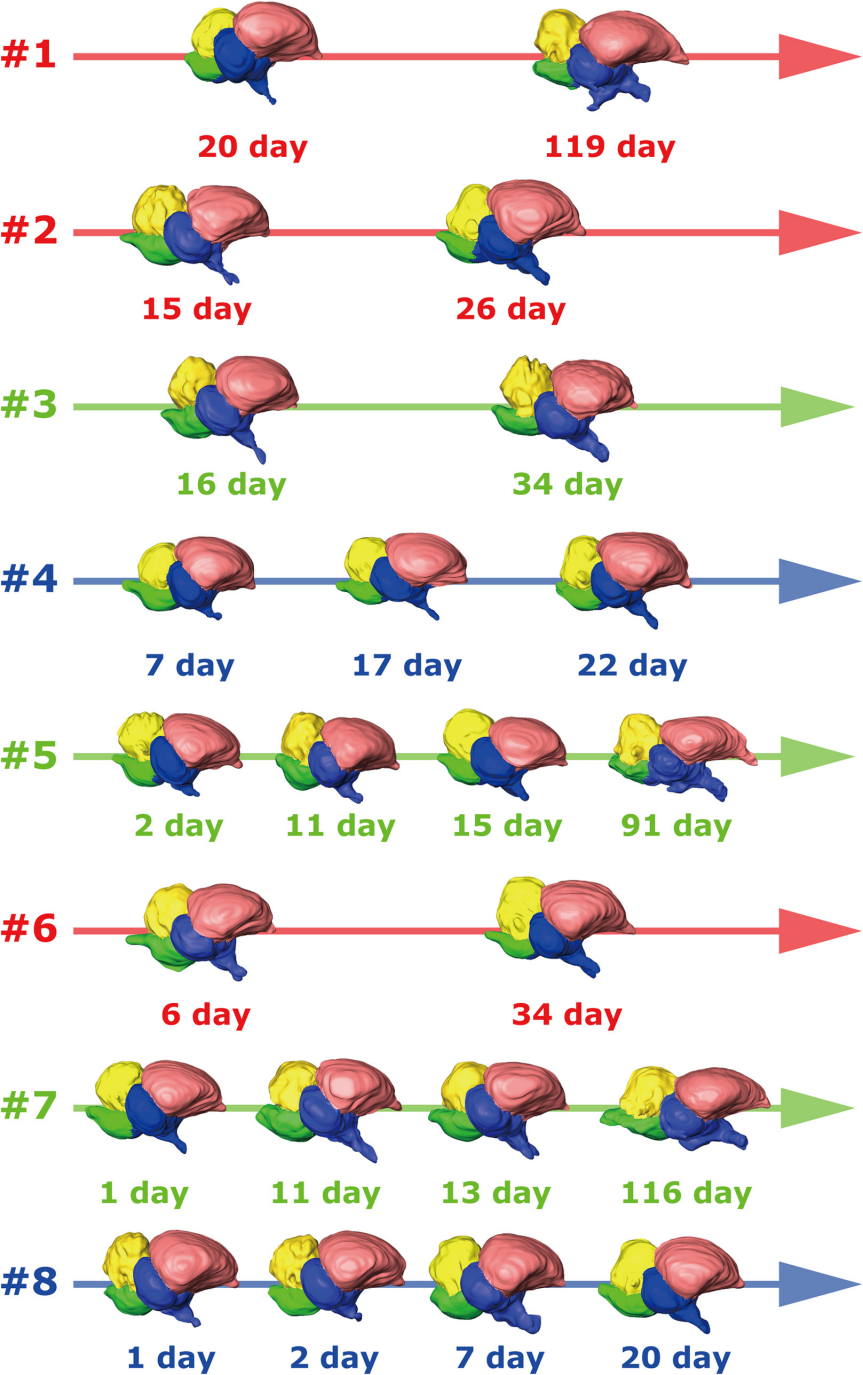
Ontogenetic shape variations (#1 to #8) (not to scale).

**Fig 4:**
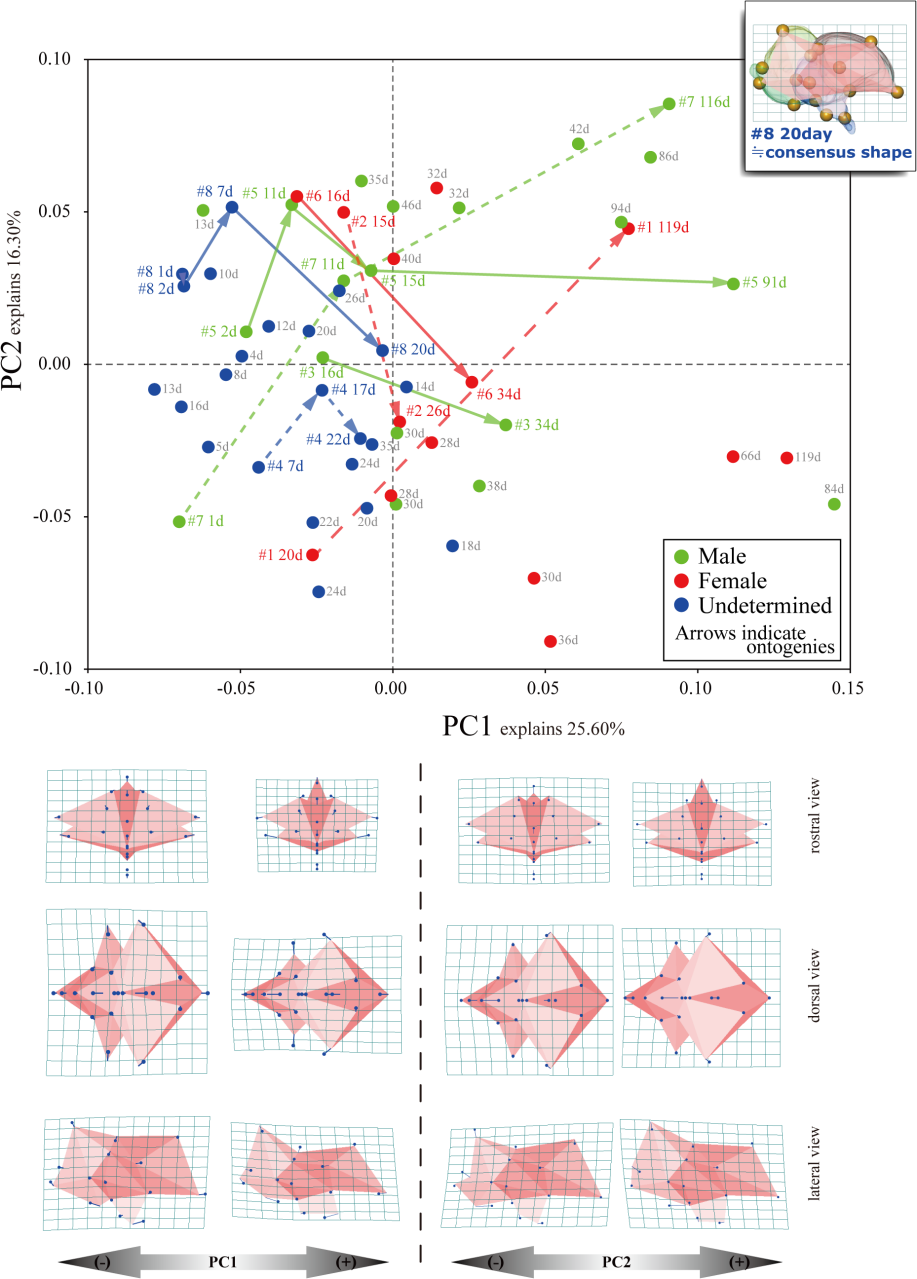
PCA and variation in brain shape for each principal component (PC) score.
